# Probable Vildagliptin-Associated Acute Pancreatitis in a Patient With Well-Controlled Type 2 Diabetes Mellitus: A Case Report

**DOI:** 10.7759/cureus.111849

**Published:** 2026-06-30

**Authors:** Kyaw Zin Aung, Naw Eh Law Saw

**Affiliations:** 1 Internal Medicine, Kulhudhuffushi Regional Hospital, Kulhudhuffushi, MDV

**Keywords:** acute pancreatitis, diabetes mellitus type 2, dpp-4 inhibitor, drug-induced acute pancreatitis, vildagliptin

## Abstract

Drug-induced acute pancreatitis (DIAP) is rare, accounting for approximately 0.1%-5% of all cases, and the association between dipeptidyl peptidase-4 (DPP-4) inhibitors and pancreatitis remains controversial despite postmarketing safety signals. We report the case of a 46-year-old man with well-controlled type 2 diabetes mellitus who presented with severe epigastric pain radiating to the back after six months of therapy with vildagliptin and metformin. Laboratory evaluation showed markedly elevated serum amylase (1,220 U/L) and lipase (2,334 U/L), exceeding three times the upper limit of normal, while contrast-enhanced CT demonstrated acute interstitial edematous pancreatitis without evidence of necrosis. A systematic evaluation excluded common etiologies, including gallstones, alcohol use, hypertriglyceridemia, hypercalcemia, pancreaticobiliary obstruction, and structural abnormalities. Vildagliptin was promptly discontinued, and the patient received supportive management, with complete clinical recovery. The temporal association, exclusion of alternative causes, and improvement following drug withdrawal (positive dechallenge), together with a Naranjo Adverse Drug Reaction Probability Scale score of 7, support a probable drug-related association, with vildagliptin considered the most likely causative agent. Clinicians should remain aware of this rare but potential adverse effect when evaluating patients receiving DPP-4 inhibitors who present with acute pancreatitis after exclusion of more common causes.

## Introduction

Acute pancreatitis is an acute inflammatory condition of the pancreas characterized by sudden-onset abdominal pain, elevation of pancreatic enzymes, and a variable systemic inflammatory response. According to the Revised Atlanta Classification, the diagnosis requires at least two of the following three criteria: typical abdominal pain, serum amylase or lipase levels greater than three times the upper limit of normal, or imaging findings consistent with pancreatic inflammation [1]. The majority of cases are attributed to gallstone disease and alcohol consumption, while less common etiologies include hypertriglyceridemia, hypercalcemia, and drug-induced acute pancreatitis (DIAP) [2,3].

Dipeptidyl peptidase-4 (DPP-4) inhibitors are widely used in the management of type 2 diabetes mellitus due to their glucose-dependent mechanism of action, favorable safety profile, and low risk of hypoglycemia [4]. However, concerns regarding pancreatic safety emerged following postmarketing surveillance reports and regulatory evaluations that identified cases of acute pancreatitis among patients receiving incretin-based therapies [5]. Despite these signals, evidence from randomized controlled trials and subsequent meta-analyses has not consistently demonstrated a statistically significant increase in pancreatitis risk associated with DPP-4 inhibitors, suggesting that any potential association is likely rare and possibly confined to susceptible individuals [6,7].

The biological mechanism underlying a possible association between DPP-4 inhibitors and acute pancreatitis remains uncertain. An idiosyncratic immune-mediated reaction has been proposed as one possible mechanism; however, clinical evidence supporting this hypothesis is limited. Another proposed mechanism is that incretin-based therapies may exert trophic effects on pancreatic ductal or exocrine tissue. However, these findings are based mainly on experimental and limited histopathological studies, remain controversial, and no definitive causal mechanism has been established in clinical populations [8,9].

Establishing causality in DIAP is inherently challenging and relies on temporal association with drug exposure, exclusion of alternative etiologies, improvement following drug withdrawal, and, rarely, recurrence upon rechallenge [3,10]. We report a case of acute interstitial edematous pancreatitis occurring after six months of vildagliptin therapy in a patient with well-controlled type 2 diabetes mellitus, in whom systematic evaluation excluded the common alternative etiologies that were clinically indicated. This case contributes to the existing literature by illustrating the application of structured causality assessment and systematic exclusion of alternative etiologies in evaluating suspected drug-induced pancreatitis associated with vildagliptin.

## Case presentation

A 46-year-old man presented to the ED with a two-day history of severe epigastric pain radiating to the back, associated with nausea and multiple episodes of vomiting. The pain was continuous, aggravated by oral intake, and rated 8/10 in intensity. He denied alcohol consumption, smoking, recent abdominal trauma, gallstone disease, fever, recent endoscopic procedures, illicit drug use, or exposure to toxins.

His medical history was significant for type 2 diabetes mellitus diagnosed six months before presentation. Glycemic control had been satisfactory, with a most recent glycated hemoglobin (HbA1c) level of 5.9%; HbA1c at diagnosis was 8.7%. Antidiabetic therapy consisted of metformin 500 mg twice daily and vildagliptin 50 mg twice daily, both initiated six months before admission.

On admission, the patient was hemodynamically stable, with a blood pressure of 120/70 mmHg, heart rate of 92 beats/min, respiratory rate of 16 breaths/min, temperature of 36.8°C, and oxygen saturation of 99% on room air. Physical examination revealed epigastric tenderness without guarding, rebound tenderness, or signs of peritonitis.

Initial laboratory investigations demonstrated a serum amylase level of 1,220 U/L (reference range: 30-110 U/L) and a serum lipase level of 2,334 U/L (reference range: 10-70 U/L), both exceeding three times the upper limit of normal. Serum triglyceride concentration was 78 mg/dL (reference range: <150 mg/dL), corrected serum calcium was 9.7 mg/dL, and liver biochemistry showed a normal pattern without cholestatic features. Renal function remained within normal limits throughout hospitalization. Serial laboratory parameters are summarized in Table 1.

**Table 1:** Serial laboratory parameters during hospitalization and follow-up. Hemoglobin (Hb), hematocrit (Hct), WBC count, and CRP were used as hematological and inflammatory markers. Serum electrolytes included sodium (Na⁺), potassium (K⁺), and calcium (Ca²⁺). Liver function parameters included alanine aminotransferase (ALT), aspartate aminotransferase (AST), alkaline phosphatase (ALP), and gamma-glutamyl transferase (GGT). Renal function was assessed using serum creatinine (Cr) and blood urea nitrogen (BUN). Arterial blood gas parameters included partial pressure of oxygen (PaO₂), partial pressure of carbon dioxide (PaCO₂), and pH.

Parameter	Day 1	Day 2	Day 3	Day 4	Day 5	Day 6	Day 7	Day 14	Reference range
Hb (g/dL)	14	13.8	13	12.8	11	11.8	11	11.2	13.5-17.5
Hematocrit (%)	43	42	40	38	34	34	32	36	36.2-46.3
WBC (×10⁹/L)	14	12	14.99	16	13	9.6	9	8.6	4-11.5
Platelet count (×10⁹/L)	347	354	250	268	241	273	304	345	150-450
Serum albumin (g/dL)	4.5	4.1	4	3.6	3.3	3.2	3	3	3.5-5
CRP (mg/L)	5	35	140	367	343	115	30	7	<10
Serum sodium (mmol/L)	136	136	135	134	141	139	138	143	135-145
Serum potassium (mmol/L)	4.1	4.6	4	3.8	3.6	3.7	3.7	3.9	3.5-5.5
Serum calcium (mg/dL)	9.7	9.5	8.8	9	8.6	8.9	9.2	9.5	8.5-10.5
Total bilirubin (mg/dL)	1.2	1.2	1.3	1.1	0.9	1	1.2	1.2	0.2-1.3
ALT (U/L)	34	38	35	40	38	38	36	38	<35
AST (U/L)	42	39	37	39	25	29	38	36	14-36
ALP (U/L)	80	83	60	90	77	109	120	98	38-126
GGT (U/L)	64	65	54	48	50	49	49	52	15-73
Cr (mg/dL)	1.1	1	1	0.9	0.8	0.8	0.8	0.8	0.52-1.04
BUN (mg/dL)	12	10	8.9	7.9	7.5	7.9	8.4	9	7-17
Lactate (mmol/L)	1.6	1.4	1.6	1.4	0.8	0.9	1.1	0.8	0.5-2.2
pH	7.44	7.42	7.34	7.44	7.42	7.39	7.41	7.43	7.35-7.45
PaO₂ (mmHg)	92	98	91	88	91	89	92	99	83-108
PaCO₂ (mmHg)	27	32	29	33	35	34	34	36	32-48
Venous plasma glucose (mg/dL)	134	98	104	122	138	124	98	112	70-180

The diagnosis of acute pancreatitis was established based on characteristic abdominal pain, markedly elevated pancreatic enzymes, and imaging findings consistent with pancreatic inflammation, fulfilling the Revised Atlanta diagnostic criteria. Severity assessment demonstrated mild acute pancreatitis, with a Bedside Index for Severity in Acute Pancreatitis (BISAP) score of 0, APACHE II score of 3, and Glasgow-Imrie score of 0. According to the Revised Atlanta Classification, the episode was classified as mild acute pancreatitis because there was no evidence of persistent organ failure, pancreatic necrosis, or organized local complications. Although serum amylase and lipase are not exclusively pancreatic enzymes and may be elevated in selected extra-pancreatic conditions, the presence of characteristic abdominal pain together with imaging-confirmed acute interstitial edematous pancreatitis fulfilled the Revised Atlanta diagnostic criteria, making an extra-pancreatic source unlikely in this case.

Abdominal USG demonstrated diffuse pancreatic enlargement with peripancreatic inflammatory changes, without gallstones, biliary sludge, or biliary ductal dilatation. Contrast-enhanced CT performed on hospital day 3 revealed diffuse pancreatic enlargement with surrounding inflammatory fat stranding and peripancreatic fluid collections, consistent with acute interstitial edematous pancreatitis, without pancreatic necrosis or organized collections (Figure 1).

**Figure 1:**
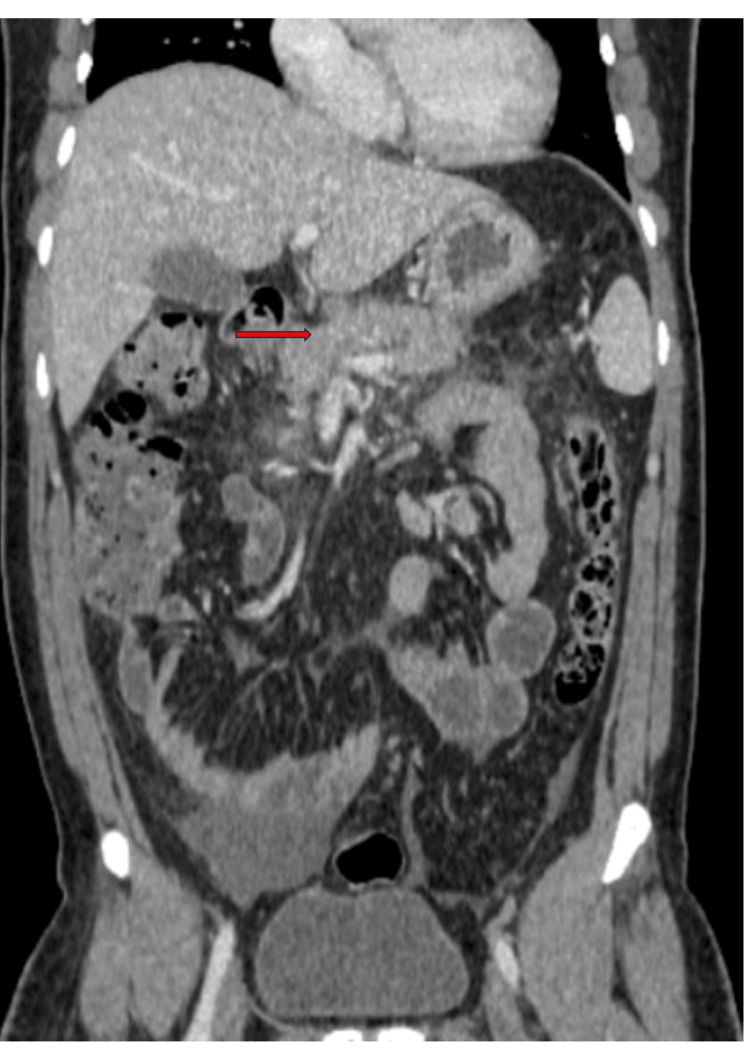
Coronal contrast-enhanced CT image showing diffuse pancreatic enlargement with peripancreatic fat stranding and fluid collections (arrow), consistent with acute interstitial edematous pancreatitis. No pancreatic necrosis or organized collections are seen.

Magnetic resonance cholangiopancreatography performed on hospital day 5 demonstrated a normal-caliber biliary tree and pancreatic duct, without choledocholithiasis, biliary obstruction, ductal stricture, or structural pancreaticobiliary abnormalities (Figure 2). No pancreatic cystic lesions or pancreatic duct abnormalities suggestive of intraductal papillary mucinous neoplasm (IPMN) were identified on CT or MRCP.

**Figure 2:**
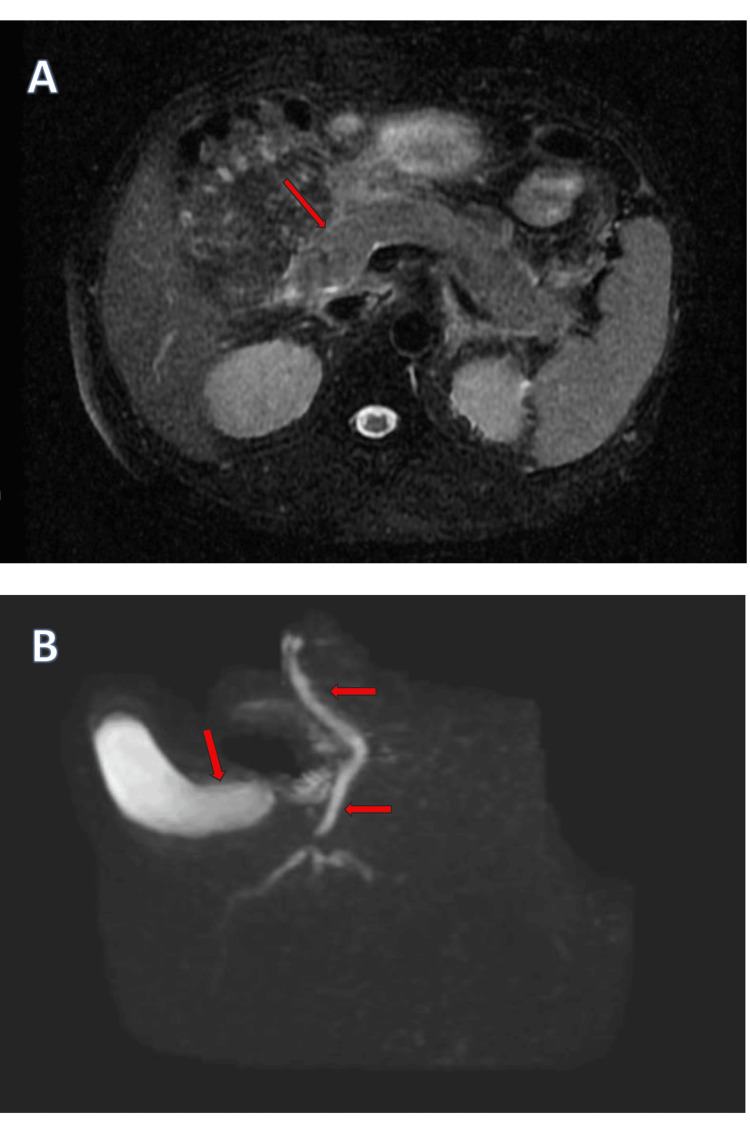
MRCP demonstrating normal-caliber biliary and pancreatic ducts without obstruction, stricture, or choledocholithiasis. (A) Axial T2-weighted image demonstrating diffuse pancreatic enlargement with surrounding peripancreatic inflammatory edema (arrow). (B) Maximum-intensity-projection MRCP image demonstrating a normal-caliber extrahepatic biliary tree (horizontal arrows) and gallbladder (oblique arrow), without evidence of choledocholithiasis, biliary obstruction, or significant biliary ductal dilatation. MRCP: Magnetic resonance cholangiopancreatography.

Alternative etiologies of pancreatitis were systematically excluded. The patient denied alcohol use, serum triglyceride and calcium concentrations were within normal limits, and imaging demonstrated no evidence of gallstones, biliary obstruction, pancreatic mass, or congenital pancreaticobiliary abnormalities. There was no history of trauma, recent endoscopic intervention, infection, or systemic inflammatory disease. Autoimmune pancreatitis was considered unlikely because there were no suggestive clinical or imaging features; therefore, serum IgG4 testing was not performed. There was also no clinical evidence of a recent viral illness to warrant virological testing.

Given the temporal association between vildagliptin exposure and symptom onset, together with the exclusion of competing etiologies, drug-induced pancreatitis was suspected. Vildagliptin was discontinued immediately upon diagnosis. The patient received IV fluid resuscitation, analgesia, antiemetics, nutritional support, and close clinical monitoring.

Clinical improvement was observed within 72 hours, with progressive reduction in abdominal pain and inflammatory markers. Serial pancreatic enzyme measurements also demonstrated progressive biochemical improvement following discontinuation of vildagliptin and supportive management (Table 2). The patient was discharged on hospital day 7 in stable condition.

**Table 2:** Serial pancreatic enzyme levels during hospitalization. Serial pancreatic enzyme levels demonstrating progressive biochemical improvement following discontinuation of vildagliptin and supportive management.

Parameter	Day 1	Day 3	Day 5	Day 7	Reference range
Serum amylase (U/L)	1,220	845	325	98	30-110
Serum lipase (U/L)	2,334	1,882	482	68	10-70

A formal causality assessment was performed using the Naranjo Adverse Drug Reaction Probability Scale. The patient achieved a total score of 7, based on previous conclusive reports (+1), a clear temporal relationship between drug administration and symptom onset (+2), improvement following drug withdrawal (+1), exclusion of alternative causes (+2), and objective confirmation of the adverse event (+1). This score indicates a probable adverse drug reaction, supporting vildagliptin as the most likely suspected agent after systematic exclusion of alternative etiologies and structured causality assessment. Drug rechallenge was not performed because of ethical concerns and the risk of recurrent pancreatitis.

Follow-up at 14 days, one month, and three months demonstrated complete resolution of symptoms, without recurrent pancreatitis or radiological complications.

## Discussion

DIAP remains a diagnosis of exclusion and requires a systematic evaluation to eliminate more common etiologies, including gallstone disease, alcohol use, hypertriglyceridemia, hypercalcemia, infection, trauma, pancreaticobiliary obstruction, and recent endoscopic procedures [2,3]. Although serum amylase and lipase are not exclusively pancreatic enzymes and may be elevated in certain extrapancreatic conditions, the combination of characteristic abdominal pain, marked enzyme elevation, and imaging findings in this patient fulfilled the Revised Atlanta diagnostic criteria, making an extrapancreatic source unlikely. In the present case, systematic biochemical testing and advanced imaging, including ultrasonography, contrast-enhanced CT, and magnetic resonance cholangiopancreatography, excluded these alternative causes, thereby strengthening the likelihood of a drug-related etiology.

The temporal relationship between initiation of vildagliptin therapy and onset of symptoms, combined with rapid clinical and biochemical improvement following drug discontinuation, supports a probable association between vildagliptin and acute pancreatitis. This is further supported by the absence of recurrence during three months of follow-up after cessation of the drug. Application of the Naranjo Adverse Drug Reaction Probability Scale yielded a score of 7, corresponding to a probable adverse drug reaction (score 5-8). Although the patient was receiving concomitant metformin therapy, metformin-associated pancreatitis is exceedingly rare and has been reported predominantly in the setting of overdose or significant renal impairment. In the present case, the patient had normal renal function, received standard therapeutic doses of metformin, and had no clinical evidence of metformin accumulation. Therefore, although the contribution of metformin cannot be completely excluded, vildagliptin was considered the more likely suspected agent based on the temporal relationship, normal renal function, therapeutic metformin dosing, exclusion of alternative etiologies, and structured causality assessment.

Concerns regarding the pancreatic safety of incretin-based therapies have persisted since postmarketing surveillance programs and regulatory agency assessments identified reported cases of acute pancreatitis among users of DPP-4 inhibitors [5]. However, subsequent randomized controlled trials and meta-analyses have generally not demonstrated a statistically significant increase in pancreatitis incidence, suggesting that if an association exists, it is rare and not clearly causal at a population level [6,7]. Therefore, current evidence supports only a weak and uncertain association rather than a definitive causal relationship.

The proposed mechanisms linking DPP-4 inhibition to pancreatic injury remain speculative. It has been hypothesized that incretin-mediated signaling may exert trophic effects on pancreatic ductal or exocrine tissue; however, these observations are primarily derived from experimental and limited histopathological studies and have not been consistently reproduced in clinical research [8,9]. As such, no definitive pathophysiological mechanism has been established, and the biological plausibility of this association remains under investigation.

Causality assessment in drug-induced pancreatitis is primarily based on clinical criteria, including temporal association, exclusion of alternative causes, and improvement after withdrawal of the suspected agent [3,10]. In this case, all major criteria were fulfilled. Although rechallenge would provide stronger evidence, it was not performed due to ethical considerations and the risk of recurrent pancreatitis.

Several limitations should be acknowledged. First, rechallenge was not undertaken, limiting definitive confirmation of causality. Second, although metformin was considered a less likely contributor based on the clinical context, its role cannot be completely excluded because the patient was receiving concomitant therapy. Third, despite extensive evaluation, an idiopathic cause cannot be entirely excluded. Therefore, the association between vildagliptin and acute pancreatitis should be interpreted as probable rather than definitive.

## Conclusions

Although uncommon, vildagliptin may be considered a possible cause of DIAP after exclusion of more common etiologies. Careful exclusion of alternative etiologies, prompt discontinuation of the suspected medication, and supportive management are essential for favorable outcomes. Increased awareness of this potential adverse effect may facilitate earlier recognition and help prevent recurrent pancreatic injury.
